# Recent Advances of Computerized Graphical Methods for the Detection and Progress Assessment of Visual Distortion Caused by Macular Disorders

**DOI:** 10.3390/vision3020025

**Published:** 2019-06-05

**Authors:** Navid Mohaghegh, Ebrahim Ghafar-Zadeh, Sebastian Magierowski

**Affiliations:** Department of Electrical Engineering and Computer Science (EECS), Lassonde School of Engineering, York University, Toronto, ON M3J 1P3, Canada

**Keywords:** macular disorders, central serous retinopathy (CSR), graphical macular interface system (GMIS), age-related macular degeneration (AMD)

## Abstract

Recent advances of computerized graphical methods have received significant attention for detection and home monitoring of various visual distortions caused by macular disorders such as macular edema, central serous chorioretinopathy, and age-related macular degeneration. After a brief review of macular disorders and their conventional diagnostic methods, this paper reviews such graphical interface methods including computerized Amsler Grid, Preferential Hyperacuity Perimeter, and Three-dimensional Computer-automated Threshold Amsler Grid. Thereafter, the challenges of these computerized methods for accurate and rapid detection of macular disorders are discussed. The early detection and progress assessment of macular disorders can significantly enhance the required clinical procedure for the diagnosis and treatment of macular disorders.

## 1. Introduction

Macular disorders such as myopic maculopathy, macular holes, diabetic macular edema, age-related macular degeneration (AMD), and central serous retinopathy (CSR) cause visual distortion (VD) in their early stages [1,2]. In their advanced phases, macular disorders cause central vision loss. Among these retinal conditions, AMD is the leading cause of blindness and will affect the central vision of 196 million people over the age of 65 worldwide by 2020 [3]. The central vision effects are very diverse and can impact patients’ basic mobility, road crossing, driving, scene viewing, reading, working with computers and electronics, and even cause depression [4,5]. Patients with macular disorders may require low vision rehabilitation assistance to achieve some simple goals like news reading, leisure and entertainment, computer/mobile use for personal communication, and correspondence. Therefore, the macular disorders severely affect the quality of life and raise the direct and indirect medical management cost of macular disorders [6]. Early detection of macular disorders is crucial as close monitoring allows for intervention before irreversible damage occurs. The earlier the VD is detected, the better the treatment of the macular disorder can be managed [7]. Currently, various imaging methods including Optical Coherence Tomography (OCT) are used as the standard assessment tools to monitor the progress of the macular disorders [8]. Although OCT is a high-resolution technique intended to show any deformation in the cross-section of the retina, OCT is not a low-cost method and only an expert can use it. Therefore, it cannot serve as a realistic choice for home-monitoring purposes. Despite significant advances in biomedical technologies, still, the early detection and home-monitoring of VD associated with various macular disorders is a significant challenge. Computerized graphical interface (CGI) methods have recently attracted eye care professionals’ attention as a low cost, home-use method for rapid detection of damage in the visual field as well as monitoring the progress of macular disorders. 

### 1.1. VD Caused by Macular Disorders

**Figure 1:**
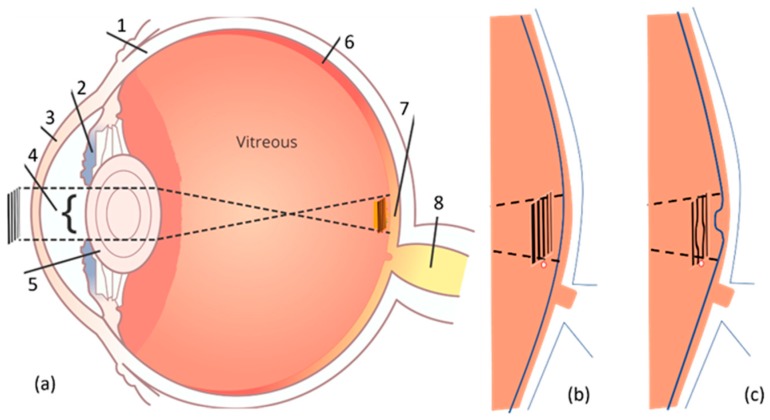
(**a**) Simplified schematic of the eye structure (**b**) healthy macula (**c**) unhealthy macula (the deformed retinal basement due to a macular disorder such as central serous retinopathy (CSR). Various parts of the eye including (**1**) sclera, (**2**) iris, (**3**) cornea, (**4**) pupil, (**5**) lens, (**6**) entire retina, (**7**) macula and in its center fovea, (**8**) optic nerve, and vitreous are shown in this figure.

As seen in Figure 1a, light passes through the cornea and the lens thus allowing an image to be focused on the retina. The retina changes this light energy into a signal that can be transmitted to the brain via the optic nerve on the top layer of the retina. The macula is a small, yellow area of the retina approximately 4 mm from the optic disk. This area contains the fovea and provides the sharpest central vision. When the gaze is fixed on an object, the macula, the lens, and the object are in a straight line. The fovea is a shallow, capillary-free, depression in the center of the macula approximately 1.5 mm in diameter and contains more than 199,000 to 300,000 cones per square millimeter [9,10]. As seen in Figure 1c, the deflection of the retina near the fovea might be due to a macular disorder such as central serous chorioretinopathy (CSR) or age-related macular degeneration (AMD) resulting in the distortion of the created image on the retina. Figure 1 shows the main components of the retina when a deflection has occurred due to a macular disorder. It is noteworthy that this deformation is the source of VD in the visual sharpness, color and even the distortion of straight lines [11,12]. 

### 1.2. Related Macular Disorders

**Figure 2:**
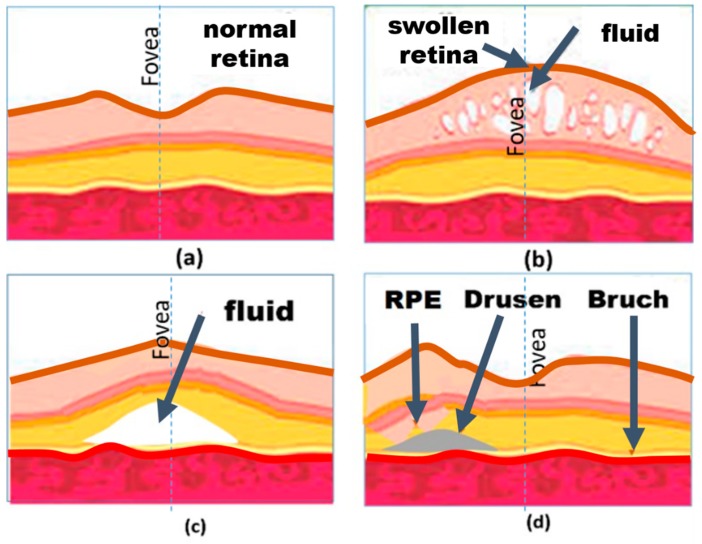
Illustration of retina section of (**a**) normal eye and the eye suffers from (**b**) edema, (**c**) central retinopathy (CSR), and (**d**) age-related macular degeneration (AMD). In this figure, RPE denotes retinal pigment epithelium.

Macular edema, CSR, and AMD are three well known macular disorders that cause deformation in the retina. This section outlines these macular disorders [1,13,14,15,16,17]. Macular edema occurs when the fluid collects within the macula leading to swelling of the retina’s tissue and subsequently results in a visual distortion [10,18]. In the retina, like other types of tissues in the body, there is extracellular fluid including water and other bio-molecules. Diabetic macular edema (DME) and cystoid macular edema (CME) are two subtypes of macular edema. DME or swelling of the retina occurs in diabetes. The leakage of fluid from blood vessels that results in this edema. In CME, fluid accumulates in cyst-like spaces within the macula. The collection of fluid or blood results in the deformation of the retina with a significant effect on vision. On the other hand, CSR is a condition that occurs because of leakage of fluid at the level of the retinal pigment epithelium (RPE) [16,17]. It is noteworthy that the epithelium is one of the four main types of tissues in the body and the RPE is the pigmented cell layer that nourishes retinal visual cells. As seen in Figure 2c, this leakage results in deformation of the retina similar to that imposed by macular edema. There is usually no underlying cause, however, in most cases the condition is preceded by work-related chronic mental stress [16,17]. In AMD, drusen—yellow colored pigments made up of lipids—builds under the basement membrane of the retinal pigment epithelium causing significant vision loss [14,15]. As the macula is the central part of the retina consisting of the highest concentration of photoreceptors, the emergence and growth of drusen over years causes loss of vision. AMD can be classified into two categories, dry and wet. In a dry AMD, drusen is small, round and discrete whereas, in AMD, drusen is larger with indistinct margins. As shown in Figure 2d, the presence of drusen can displace the mosaic retina causing patients to complain about the observation of wavy lines [19,20]. 

In addition to AMD, CSR, and macular edema, other types of macular disorders may result in the deformation of the retina and thus VD in the early stages of the development of the disease [10,12]. 

### 1.3. VD Assessment in Patients with Macular Disorders

**Figure 3:**
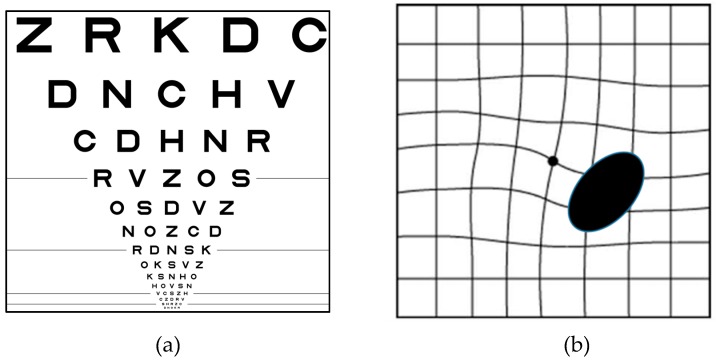
Visual chart tests: (**a**) ETDRS Visual Acuity Chart [21] (Reproduced with permission from Wolters Kluwer Health, Inc.) and (**b**) Amsler grid with Scotoma (right) and Metamorphopsia (left) seen on Amsler grid in AMD patients [10,26] (Reproduced with permission from Springer Nature and BMJ respectively).

Among various visual assessment methods, the visual acuity test [21] is widely used to evaluate the smallest letters that a patient can read on a standardized Snellen chart or particularly Logarithm of the Minimum Angle of Resolution (LogMAR) chart as displayed in Figure 3a. Despite the great advantages of ETDRS, the studies show that visual acuity is a poor indicator of macular disorders including AMD [22,23,24]. This is because the acuity test can only evaluate the presence of AMD, but it does not provide any information about the location or the progression of AMD. 

In 1947 Marc Amsler created a printed grid for the detection of AMD, which he called the Amsler Grid [25]. Currently, the Amsler Grid (AG) is the gold standard of home monitoring for patients suffering from macular disorders. The grid is used in a monocular fashion with their best near correction (see Figure 3b) while the participants are asked to fixate at the center. An AG sheet approximately 8 × 8 inches in size should be held 20 cm from one eye when the second eye is covered. In this test, first, the patients should wear their eyeglasses and look at the dot in the center of the AG sheet. Then, they should detect any missing corners or any lines that are wavy or missing. The AG test can successfully monitor metamorphopsia and scotomas as seen in Figure 3b [24]. Although it is widely used, the AG shows low accuracy for detecting VD changes smaller than the distance between the horizontal or vertical lines in patients suffering from AMD from one time to another. Moreover, AG has limitations such as cortical completion (the brain completes a partially formed image) [26], lack of accurate central fixation (looking at an object involves our macula and is called central fixation), crowding of the lines (confusion due to the presence of multiple lines), and poor patient compliance (there is no way to monitor if they actually did the test every day).

OCT is a noninvasive medical imaging technique used to provide an optical cross-section of the retina. This technique is widely used for obtaining sub-surface images of opaque materials at a resolution equivalent to a low-power microscope. By employing near-infrared light, it can capture micrometer-resolution features [10,27].

OCT can play a key role in evaluating patients with macular disorders and those undergoing treatment [28]. Proper analysis of OCT images requires training and extensive practice, for this reason, it is not suitable for home diagnostics. Figure 2a–d shows OCT illustrations of normal, AMD, and macular edema afflicted retinas [29,30].

As aforementioned computerized graphical VD assessment methods have recently emerged to overcome the problems. Despite significant advances, there are many challenges in developing and using computerized VD assessment techniques. These methods can be divided into three main groups including static, dynamic and movable approaches that are put forward in Section 2 and Section 3 respectively. Also, the challenges of computerized methods are discussed in Section 4 followed up with a conclusion in Section 5. Table 1 shows the frequently used acronyms in this paper.

## 2. Static VD Assessment Methods

As aforementioned, AG is used as a standard method to detect scotoma and metamorphopsia. In this method, the grid pattern is used as a still image while the patient fixates at the center of the display and tries to detect any deformation or curvy lines in the grid. 

### 2.1. Enhanced AG Tests

**Figure 4:**
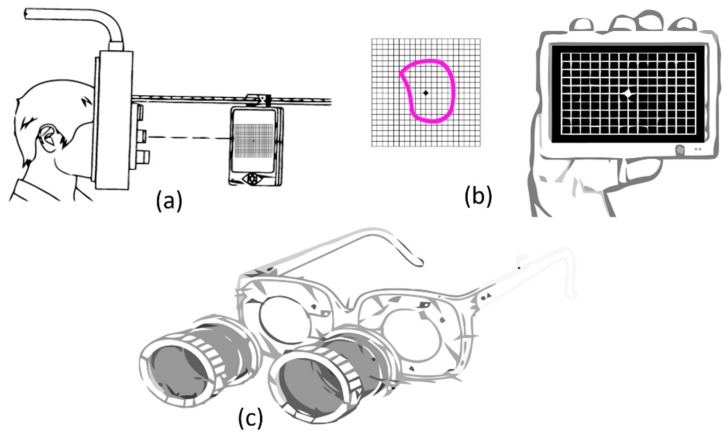
(**a**) Near eye ophthalmic device utilized with a phoropter rod to better display Amsler grid, (**b**) Portable smart tablet used for evaluation of metamorphopsia, and (**c**) Eyeglasses used in threshold Amsler grid test equipped with mountable polarized filters.

Many efforts have been made to convert the paper-based AG test to a computerized AG test [31,32]. Among these efforts, as shown in Figure 4a, Hirji [33] presented a portable near-eye ophthalmic device using a portable tablet computer incorporated with a chinrest-like phoropter rod to limit the movements of the head and allow a better view of the Amsler Grid. Despite the fact that this device allows for better fixation at the center of the grid by reducing the head movements, as with the AG charts’ test, it still suffers from the completion effect. As described in [34], the brain fills or completes the gaps in the visual field of each eye [35]. Therefore, this completion effect may cause wrong answers in this test. As shown in Figure 4b a finger-touch tablet or smartphone can be used for directly marking any area of VDs such as metamorphopsia. By offering advantages of faster interaction with patients, this technology not only can be used in eye-care clinics but also, a portable smart tablet is suitable for self-monitoring of macular disorders at home. Despite the advantages mentioned above, this method, in addition to its problems with the completion effect, also suffers from a lack of eye-tracking to assure fixation compliance during testing. 

### 2.2. Threshold Amsler Grid Test

As described in sub Section 2.1, the AG is directly displayed in front of the eye. Therefore, the accuracy of detecting scotoma and metamorphopsia is limited by overall visual acuity and the performance of the eye in functioning properly or not. The Threshold Amsler Grid (TAG) test has been designed to increase the accuracy of the traditional AG tests. A TAG test can be performed through cross-polarizing filters that cause low luminance conditions in which the grid becomes barely perceptible as seen in Figure 4c [36]. It is noteworthy that the cross-polarizing filter is used for enhancing vision by filtering the reflected lights. However, this filter reduces luminance conditions. Such eyeglasses, reported by Sadun et al., can vary the amount of luminance of the observed AG by the patient [37]. Therefore, this will greatly increase the sensitivity of the patient’s eyes to perceive the presence of scotomas. Although the threshold Amsler grid shows better performance in comparison with traditional AG method, 50% of all scotomas remain undetected [38]. 

### 2.3. Accelerated Amsler Grid Test

**Figure 5:**
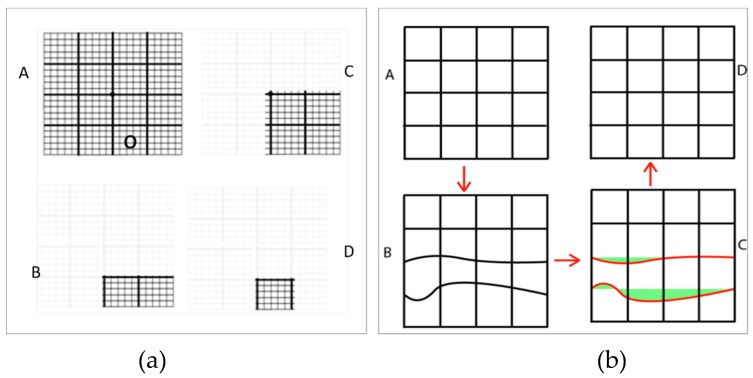
Amsler Grid (AG)-based VD detection methods (**a**) Binary Amsler Interactions (**b**) Deformable Amsler Grid (DAG). DAG has ability to apply correction vectors to improve patient vision (A) denotes the normal grid (B) is the grid as seen by the patient (C) is the correction vectors that are highlighted in red and correction values that are measured in green which denotes the amount of deflection from the straight grid lines (D) is the improved final grid as viewed by the patient after applying the correction vectors.

In an AG test, the patients should explain their VD experience in order to approximately find the locations of VD in their visual fields, however, with AG test, patients cannot be provided with a quantitative analysis of their VD. One of the first steps towards quantifying these answers was taken by proposing the accelerated AG test. In this test, sub AG blocks ⅟₄, ⅟₁₆, and ⅟₃₂ the size of the largest AG block (with 32 small blocks like C in Figure 5) are displayed for covering the entire display (A, B, and C in Figure 5a). Patients provide ‘yes’ or ‘no’ answers in response to viewing these blocks. The observation of a normal block with straight-lines or abnormal blocks with curvy-lines can represent the presence or absence of VDs respectively. In this method, proposed by Palanker [39], at the first step, AG block A is displayed in the right-top, right-bottom, left-top, and left-bottom in order to detect the location of VD. In the next steps, the AG blocks C and D are scanned in the detected location with VD in order to accurately detect the location of VD. The accuracy of this method is limited to the distance between the lines that create sub AG blocks. Figure 5 shows that VD can be observed in two adjunct blocks or other combinations of the smallest AG blocks. In this method, the smallest detectable metamorphopsia lesions in the visual field of the patient can be equal to the smallest AG block C. By decreasing the size of the smallest block, for instance to ⅟₆₄ or lower, the accuracy of this technique is increased, however, it will require more time to scan all blocks thus causing eye fatigue. Consequently, it results in higher difficulty to fixate on the center of the screen.

By assuming a VD happens in sub AG block D (denoted with “o” in Figure 5a-A, after answering seven “yes/no” questions; the VD block can be identified. One may suggest a pure binary search tree approach to accelerate the speed even further. Despite the advantages of this method, this method still may suffer from the completion effect and a lack of controlled fixation which are inherited from the Amsler Grid as already explained. 

### 2.4. Deformable Amsler Grid

This method relies on the fact that when an AG with all straight lines (Figure 5b-A) is projected on the patient’s retina, and the patient partially see a deformed (Figure 5b-B) line. Assuming that the patient can spot the metamorphopsia displayed in Figure 5b-B, then it could be possible to project an AG with the same deformed lines (Figure 5b-C) but in the opposite direction to ease the metamorphopsia experienced by the patient as shown in Figure 5b-D. As shown in Figure 5b-C, the alternate grid has the same deformed lines, but in the opposite direction. This is achieved by applying a correction vector (highlighted in red in Figure 5b-C). Figure 5b-D is the result of the correction vector being applied. 

Based on this method, Kohn et al. proposed [40] a portable device including a tablet with a deformable AG program that can interactively measure the metamorphopsia via measuring the correction vector. Based on this technique, the patient can change the location of the vertices of the grid until the grid appears substantially rectilinear to the patient. The deformations of the pattern are recorded by the program and can be retrieved for evaluation of deformation and the progress of disorder. The inherited difficulties of the AG test such as the completion effect apply to this technique as well. Additionally, the fixation at the center is another important problem; This is because the patient’s focus will move toward the deformation location while interactively changing the deformations.

### 2.5. NGRID VD Method

The static VD assessment methods suffer from the filling and crowding effect due to the similarity of stimulation patterns. We have recently addressed this issue by proposing a novel method called NGRID [41,42,43]. In this method, instead of one grid such as Amsler grid or another type of pattern, a number of different patterns are projected on the patient’s retina. Due to the difference in patterns, the filling effect might be reduced. On the other hand, the proposed NGRID method, by taking the advantage of voice recognition for collecting the responses, has significantly increased the accuracy of measurement by eliminating any pointing tasks to assure better fixation at the center of the screen. The responses are used to estimate the location of VD in the visual field. The proposed platform allows patients to access the software for running the test and to securely store the data for professional follow-up as well as affording the possibility of consistently evaluating the progress of the disorder over time. 

## 3. Dynamic VD Assessment Methods

The second group of VD assessment methods relies on projecting the stimulation pattern over a short time and indirectly measuring the visual performance based on the ability of the eye in detecting the stimuli. These methods are dynamic in nature. It is noteworthy that the projection time of graphics to patients’ retina in dynamic methods is much shorter the static methods. 

### 3.1. Preferential Hyperacuity Perimeter (PHP) Test

Preferential Hyperacuity Perimeter (PHP) is another computerized VD assessment method that utilizes hyperacuity to detect changes and distortions in the central retina. Hyperacuity is the ability to detect small misalignments between two lines. This acuity-based measurement method is found to be much more accurate than Snellen type acuity (see chapter 1) VD detection in patients suffering from macular disorders [38,44,45]. Based on the literature, hyperacuity can also be defined as the capability of the visual sensor to transcend sampling limits set by discrete receiving elements.

**Figure 6:**
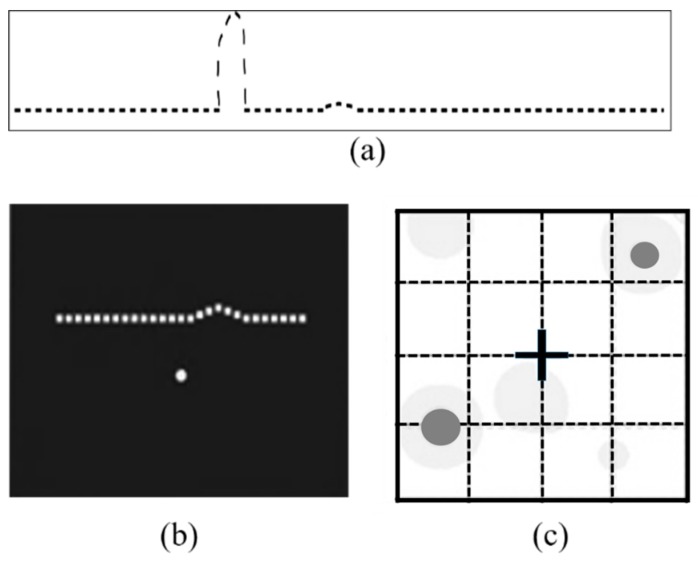
(**a**) Presenting a dotted line with the presence of a small bump and another large bump for 500ms to healthy individuals. (**b**) Preferential Hyperacuity Perimeter (PHP) test apparatus—a dotted line with a very small artificial bump shortly flashed on the screen and patients should record where they see a bump using a stylus pen [46] (**c**) is the generated PHP Heatmap that shows the location and severity of visual distortions in patient’s visual field [47]. Reproduced with permission from Wolters Kluwer Health, Inc.

As seen in Figure 6a, if a dotted line with a small bump and a large bump (one bump is very small in comparison to the other one) is presented to an individual for 500 ms, the individual may not be able to perceive the smaller bump due to the presence of the larger bump. PHP relies on this concept and simply flashes a dotted line across the screen for a short period (Figure 6b). This dotted line contains a small visual distortion (e.g., a bump in the line that can be as small as 0.3 degrees). Patients with a macular disorder might ignore this small bump due to the presence of a bigger VD caused by the macular disorder. Patients should record any perceived distortions by clicking the “bump” they have observed on the screen. A macular map, also known as a Heatmap, (Figure 6c) is obtained with quantitative values of the area and intensity of the metamorphopsia. The higher the number of clicks around the areas that no artificial bump is presented, the higher the chance of metamorphopsia presence in those regions. 

### 3.2. D-CTAG Test

**Figure 7:**
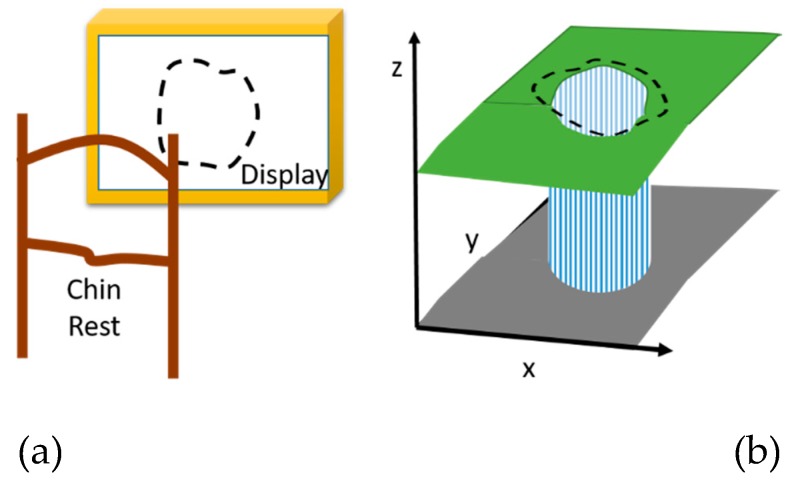
3D-CTAG Apparatus presented by Robison et al. [48]. Reproduced with permission from BMJ. (**a**) shows the overall apparatus with chin rest to limit the head movements (**b**) final result of the test which highlights the severity of affected visual distortion areas.

Three-dimensional Computer-automated Threshold Amsler Grid (3D-CTAG) is another visual field test [31,48]. Patients, with one eye covered, are positioned in front of a touch-sensitive computer screen on a head-chin rest. They are asked to finger-trace the areas of an Amsler grid that are missing from their field of vision (Figure 7a). Various degrees of contrast of the Amsler grid are presented by repeating the test at different grayscale levels. It is noteworthy that the number of vertical and horizontal lines and their width can be changed to achieve various contrasts. 

3D-CTAG is used to test the 25 degrees of the central visual fields at different contrast levels. Among the efforts made in this direction, Robison et al. reported a 3D-CTAG test with five different contrast levels [48] (5%, 10%, 20%, 40%, and 100% contrast). The detected VD area with each contrast was mapped to one of five different levels in the *z*-axis direction denoted with colors in Figure 7b. This method can reflect the effect of contrast on the accuracy with which a VD area is detected; however, still, the visual fixation at the center is a problem. This is because the attention of the patient jumps from the center of the display to its surrounding area as shown in Figure 7a.

### 3.3. Macular Computerized Psychophysical Test

**Figure 8:**
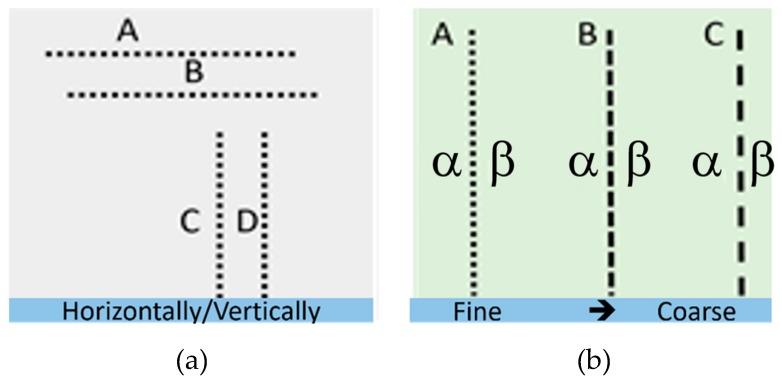
Stimulating patterns used in (**a**) Macular Computerized Psychophysical Test (MCPT) and (**b**) M-Chart test techniques.

Macular Computerized Psychophysical Test (MCPT) is another visual field test that acts based on hyperacuity [38,49]. The patient has to bring the mouse cursor to the center point on the screen to view the flashed dotted line. This task initiates a stimulus and simultaneously a forced fixation. This task is called Task Oriented Fixation (TOF) and should be repeated in order to view the next flashed dotted-line. This TOF based test ensures that the patient is going to re-fixate at the center of the screen even in the event that they momentarily lose their central fixation. A dotted line (white dots on a black background with maximal contrast) flashed at a random order across different perifoveal locations (see Figure 8a). Using a dotted line (line A, Figure 8a), the test is similar to the aforementioned hyperacuity assessment methods in accurately detecting the VDs. In MCPT the patient uses a mouse to click on a central dot, after which a new dotted line is presented at a different location line B, Figure 8a on the screen. The patient marks any scotoma or metamorphopsia by marking the corresponding locations on the screen with a mouse (C, D, Figure 8a) [38]. 

### 3.4. M-CHART Score Test

A metamorphopsia chart or so-called M-CHART is used to quantify the metamorphopsia in patients’ visual field [50,51]. The test contains 19 horizontal and vertical dotted lines with different widths and lengths that are displayed in different visual angles ranging from 0.2 to 2.0 degrees. The lines are presented at 30 cm, monocularly, while patients are allowed to have their eye-glasses to achieve best near corrected vision. As shown in Figure 8b, the patient starts by seeing a vertical (A, B, C, Figure 8b) or horizontal dotted line in between two letters (α, β, Figure 8b) that is seen by the patient as curved or misaligned. The patient is then presented with changes in the dots from fine to coarse until the metamorphopsia disappears. The minimum visual angle needed to detect the metamorphopsia is recorded [52]. In this technique, the fixation at the center of the visual field is not trivial. Additionally, it is challenging to obtain metamorphopsia scores from those patients with visual acuities 20/100 or less or the ones suffering from very large central scotomas.

### 3.5. Shape Discrimination Test 

**Figure 9:**
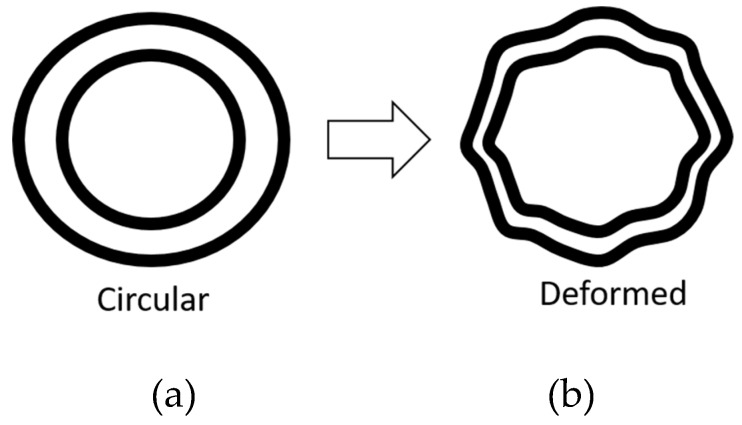
Shape Discrimination Test (**a**) circular pattern and (**b**) another similar pattern with radial deformation of 8/2π (right) [53]. Reproduced with permission from Elsevier.

Wang et al. [53] proposed a method to test visual hyperacuity using shape discrimination. In this test as seen in Figure 9a,b, one circular pattern is shown to the patient along with another similar shape that is deformed slightly. Patients should identify the deformed pattern. The test showed that AMD patients have significantly worse performance in detecting radial deformation of the patterns when compared with normal control eyes [53]. The primary goal of this test is the early detection of a macular disorder. Very small circles can be screened in the patient’s retina in order to quantify the progress of the macular disorder. However, the smaller the circles, the lower the accuracy in distinguishing normal from deformed circles. 

### 3.6. Positioning Techniques

**Figure 10:**
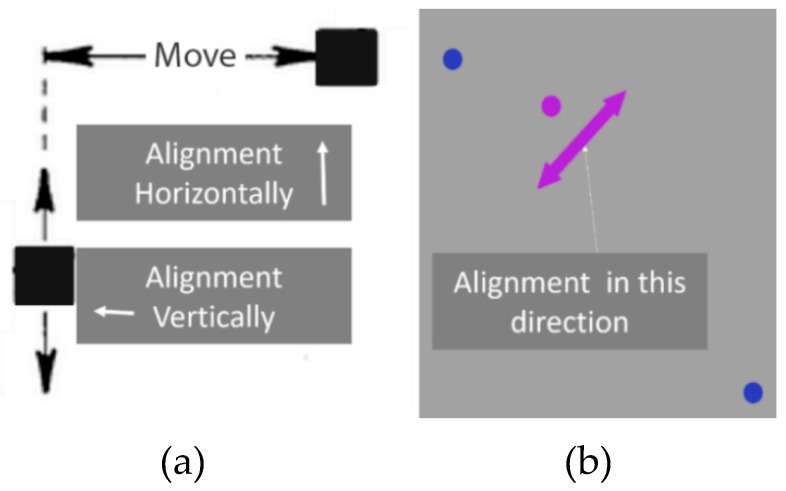
Aligning Method: (**a**) Basic Aligning Task and (**b**) Vernier Hyperacuity Test.

**Figure 11:**
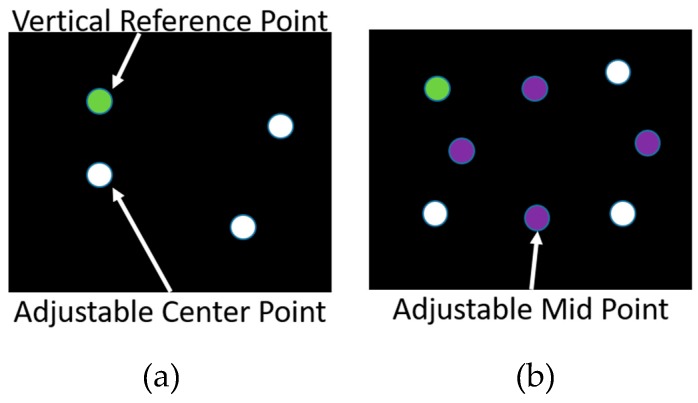
Square completion task: (**a**) a fixed green dot and three adjustable white dots and (**b**) four movable orange-colored bisecting dots [55]. Reproduced with permission from ARVO/IOVS.

**Figure 12:**
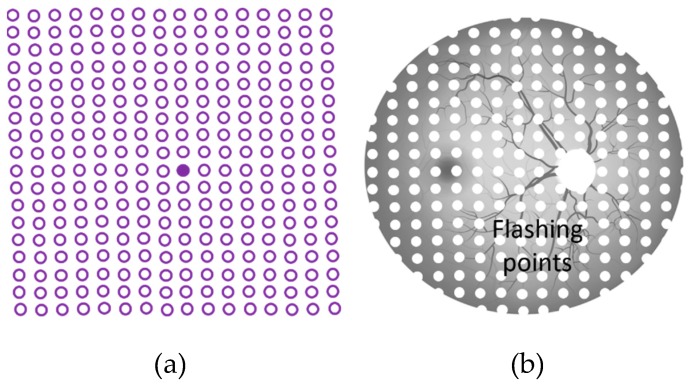
Vibrating methods: (**a**) Oscillating visual stimuli [56] and (**b**) real-time retinal tracking [57] (Reproduced with permission from ARVO/IOVS).

There were many moveable tasks introduced over the years to assess the visual field. For instance, Enoch et al. [54] reported a method for measuring metamorphopsia. As shown in Figure 10a, while one of the blocks is fixed, the other block is movable, and the patient should be able to align it vertically or horizontally with another one. In another effort, Schmid et al. presented the Vernier Hyperacuity Test (see Figure 10b), with a black background screen and a few white objects that are slightly misaligned and should be aligned by the patient. A more complex aligning task was proposed by Weicek et al. [55] who reported the square completion task with movable and immovable dots. The patient should be able to move the dots in order to form a rectangular shape as shown in Figure 11a and Figure 12b. The patient should be able to detect the dots and accurately align them horizontally and vertically. In all the above-mentioned tests with movable patterns, still it is difficult for patients to maintain fixation at the center of the screen. Stewart [56] addressed this problem by proposing oscillating visual stimuli (OVS) to detect metamorphopsia. The test pattern (see Figure 12a) is positioned in front of the patient to cover at least 40° or more of the visual field. 

Similarly, in the real-time retinal tracking method [56], a random flash point is projected in a different location of the patient’s retina (see Figure 12b). The patient should immediately press a button once a projected flashed light point is seen. The method is widely used for eye diseases such as glaucoma [57]. It is noteworthy that the aforementioned methods are used to detect the locations of VD in the visual field and to consequently detect metamorphopsia.

## 4. Discussion

Computerized static VD assessment methods including portable threshold, accelerated, and deformable AG methods, in general, are prone to the filling-in effect and may not detect small metamorphopsia and scotoma regions. This is due to the predictive shape of the grid and the fact that the brain will guess and fill-in the gaps. As reported by Fink et al. [37] 3D-CTAG like other AG based methods suffer from the filling in effect due to the predictable static grid-base nature of the test. This effect was addressed in the NGRID method [42] by offering a series of different patterns projected in a different location of the retina. Additionally, AG methods slightly suffer from the inaccurate fixation at the center of the screen if they are used as home-monitoring tools, however, if the collection of responses are managed by an assistant, the fixation at the center can be better performed. This is because the attention of the patient will be placed at the center during the test.

In comparison with the aforementioned AG-based methods, by taking the advantages of hyperacuity, PHP allows higher accuracy so that this method enables the detection of small metamorphopsia and scotomas in the early stages of development of these disorders [58]. Although PHP demonstrates high accuracy in comparison with AG methods, it allows very weak fixation at the center of the screen. This is because the locations of the bump shape patterns are randomly changed and the patient should detect them using a mouse or finger-touch screen. Using dynamic methods such as PHP, the entire central field should be covered. In other words, although PHP, M-CHART, and Shape Discrimination all quantify metamorphopsia, none of these tests track eye movement and therefore cannot ensure proper fixation during the test [2,59,60,61]. Weicek et al. [55] used a computer-based Amsler grid test, square completion, and dioptric pointing task with eye-tracking to quantify metamorphopsia in patients with maculopathy.

Similar to PHP, M-Chart lines also have to scan of view to be able to detect metamorphopsia and scotomas accurately. However, this increases the period of the test and subsequently decreases the accuracy. Furthermore, each line in the M-Chart should be repeated multiple times to cover from fine to coarse dotted lines. For MCPT with a flash duration of greater than 180 ms, the dots located on a retinal lesion theoretically should be perceived as being either misaligned or missing. This is because, for a short duration, the fovea requires re-fixation on the displayed line and this shift in fixation should cause the apparent movement of these dots from a misaligned position to an aligned one, thus giving the patient the perception of movement. For flash durations less than 180 ms, such apparent movement could be extinguished and the sensitivity of the test reduced [38]. One may argue the static tests such as AG based methods not only lower diagnostic accuracy due to poor compliance in fixation and inherit crowding and filling-in effects [62], they cannot be quantified to show the progress of disorders. On the other hand, dynamic tests such as PHP and MCPT, by offering higher accuracy, can be considered as better choices to quantify the progression of macular disorders. It is noteworthy that moveable methods, such as the Square completion task and Oscillating Visual Stimuli can be considered in the group of dynamic methods with higher accuracy. 

Table 2 presents a summary of GI methods, their advantages and disadvantages, and other details based on our study in this paper. Table 2 compares various factors including the (1) accuracy, (2) complexity or easy-to-use, (3) required time for a complete test, (4) portability. As seen in this table, as per the discussions, “High”, “Low”, and “Medium” have been used to compare factors 1–4. As also seen in this table, in all methods except AG, threshold AG and PHP home, PC should be employed. Therefore, they are considered as portable methods. However, by developing the smart-phone version of these methods their portability can become ‘good’. Another factor is complexity. A comparison between the complexity of patterns projected in the patient’s retina, shows that in AG and Accelerated AG, and Shape Discrimination methods simple patterns are used and accordingly detect the location of VD through a low complexity procedure. This complexity can increase the required time to run a test. The required time for running a simple test such as AG or threshold AG is low. However, these methods cannot be used for quantifying the progress of macular disorders and, also, they can not necessarily be used to accurately detect them. Among various factors, as per the literature, PHP method has demonstrated higher accuracy.

**Figure 13:**
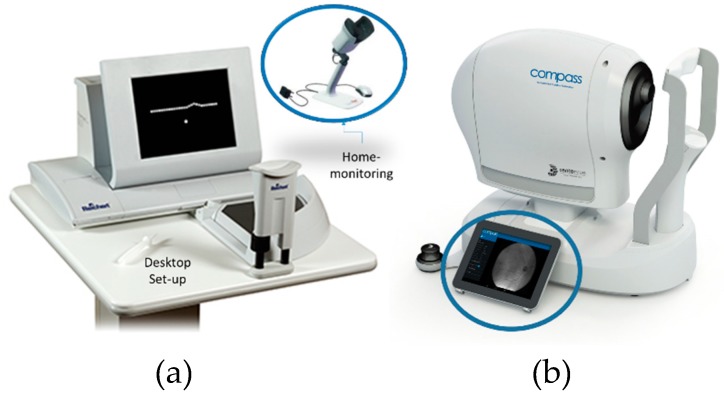
Commercially available visual field monitoring systems using (**a**) Preferential Hyperacuity Perimeter (PHP) method with two versions of desktop and portable devices and (**b**) real-time retinal tracking method.

All aforementioned visual assessment methods have great potential for commercialization by offering new software applications to run in the PC, laptop, or smartphone. However, for highly professional visual distortion assessment with high accuracy, the design and implementation of a specific device is required. In this direction, the PHP method was commercialized by Foresee Inc [63], [64,65,66] for monitoring the macular disorder (see Figure 13a). Another company called Centervue [67] developed a device for monitoring the visual field using various methods including real-time retinal tracking (see Figure 13b). Despite significant advantages, these devices are expensive and can be suitable for professional eye-care settings. It is worth mentioning that while the fixation problem could be mitigated in the future by using an eye tracker, most commercially or clinically available tests have not included the eye tracker system in their measurement instruments. Additionally, there are many psychological or physiological factors that can affect the performance of proposed computerized methods. Therefore, the success of these methods is dependent on the clinical trial. To date a few efforts have been made to systematically study the performance of graphical interface techniques for various macular diseases. Among various techniques discussed in this paper, only AG and PHP have been systematically studied and their statistics widely reported based on various clinical trials. As reported in [68,69], the sensitivities of AG and PHP for the detection of macular AMD are 78% and 87% respectively. It is noteworthy that the focus of this paper was placed on the graphical interface methods from a computer science perspective. This paper is the first to review these methods. Most of the proposed methods discussed in this review need to be used by experts and trained personnel. Therefore, in their current form they are not suitable for home diagnostics. However, these methods potentially can be used at home in the future using, for instance, a smart phone and tablet. The advantage of these methods for home diagnostic purposes have been reported in other review papers [68,69]. The focus of this paper was placed on the graphical computerized methods used for the detection of macular disorder.

## 5. Conclusions

In this paper, we put forward graphical interface methods for the assessment of the visual field. We discussed the challenges in developing accurate, rapid, and home monitoring techniques. The static methods such as Amsler Grid portable, deformable, accelerated, and threshold-based approaches were introduced. For AG-based methods, the filling-in effect and other associated issues were discussed. Also, dynamic methods such as PHP, M-Chart, MCPT, and Shape Discrimination were presented, and their advantages and disadvantages in comparison with static methods were discussed. The computerized methods can be used for home monitoring of macular disorders. This significantly helps to manage the clinical procedures and consequently improve the quality of life in patients. 

## Figures and Tables

**Table 1:** Frequently used acronyms.

Term	Acronym
Central Serous Retinopathy	CSR
Age-related Macular Degeneration	AMD
Retinal pigment epithelium	RPE
Diabetic Macular Edema	DME
Amsler Grid	AG
Optical Coherence Tomography	OCT
Logarithm of the Minimum Angle of Resolution	LogMAR
Visual Distortion	VD
Threshold Amsler Grid	TAG
Preferential Hyperacuity Perimeter	PHP
Three-dimensional Computer-automated Threshold Amsler Grid	3D-CTAG
Macular Computerized Psychophysical Test	MCPT

**Table 2:** A quick comparison of visual distortion methods.

Test	Accuracy	Complexity	Measurement Time	Portability	Ref.
**Amsler Grid (AG)**	Low	Low	Low	Good	[25,31]
**Threshold AG**	Medium	Low	Low	Medium	[38]
**PHP**	High	Medium	High	Poor	[38,44,45]
**PHP Home**	High	Medium	High	Good	[63,64,65,66]
**3D-CTAG**	Medium	Medium	High	Poor	[31,48]
**MCPT**	Medium	High	High	Poor	[38,49]
**M-CHART**	Medium	Medium	Medium	Poor	[50,51]
**Shape Discrimination**	Medium	Low	Medium	Poor	[53]
**Positioning**	Medium	High	High	Poor	[54]

## References

[1] (2004). The Eye Diseases Prevalence Research Group, Prevalence of age-related macular degeneration in the United States. Arch. Ophthalmol..

[2] Wang M., Munch I.C., Hasler P.W., Prünte C., Larsen M. (2008). Central serous chorioretinopathy. Acta Ophthalmol..

[3] Wong W.L., Su X., Li X., Cheung C.M.G., Klein R., Cheng C.Y., Wong T.Y. (2014). Global prevalence of age-related macular degeneration and disease burden projection for 2020 and 2040: A systematic review and meta-analysis. Lancet Glob. Health.

[4] Taylor D.J., Hobby A.E., Binns A.M., Crabb D.P. (2016). How does age-related macular degeneration affect real-world visual ability and quality of life? A systematic review. BMJ Open.

[5] Bressler N.M., Bressler S.B., Fine S.L. (1988). Age-related macular degeneration. Surv. Ophthalmol..

[6] The Lancet (2018). Age-related macular degeneration: Treatment at what cost?. Lancet.

[7] Busch C., Wakabayashi T., Sato T., Fukushima Y., Hara C., Shiraki N., Winegarner A., Nishida K., Sakaguchi H., Nishida K. (2019). Retinal Microvasculature and Visual Acuity after Intravitreal Aflibercept in Diabetic Macular Edema: An Optical Coherence Tomography Angiography Study. Sci. Rep..

[8] Spaide R.F., Fujimoto J.G., Waheed N.K., Sadda S.R., Staurenghi G. (2018). Optical coherence tomography angiography. Prog. Retin. Eye Res..

[9] Rogers A.H., Duker J.S. (2008). Retina.

[10] Holz F.G., Spaide R.F. (2010). Medical Retina: Focus on Retinal Imaging.

[11] Midena E., Pilotto E. (2017). Microperimetry in age: Related macular degeneration. Eye.

[12] Midena E., Vujosevic S. (2015). Metamorphopsia: An Overlooked Visual Symptom. Ophthalmic Res..

[13] Besharse J.C., Bok D. (2011). The Retina and Its Disorders.

[14] Sarwar N., Gao P., Seshasai S.R., Gobin R., Kaptoge S., Di Angelantonio E., Ingelsson E., Lawlor D.A., Selvin E., Emerging Risk Factors Collaboration (2010). Diabetes mellitus, fasting blood glucose concentration, and risk of vascular disease: A collaborative meta-analysis of 102 prospective studies. Lancet.

[15] Bourne R.R., Stevens G.A., White R.A., Smith J.L., Flaxman S.R., Price H., Jonas J.B., Keeffe J., Leasher J., Naidoo K. (2013). Causes of vision loss worldwide, 1990–2010: A systematic analysis. Lancet Glob. Health.

[16] Spaide R.F., Campeas L., Haas A., Yannuzzi L.A., Fisher Y.L., Guyer D.R., Slakter J.S., Sorenson J.A., Orlock D.A. (1996). Central serous chorioretinopathy in younger and older adults. Ophthalmology.

[17] Peiris T.J., El Rami H.E., Sun J.K. (2018). Central serous chorioretinopathy associated with steroid enema. Retin Cases Brief. Rep..

[18] Schlote T., Grueb M., Mielke J., Rohrbach J.M. (2011). Pocket Atlas of Ophthalmology.

[19] Hogg R.E., Chakravarthy U. (2006). Visual function and dysfunction in early and late age-related maculopathy. Prog. Retin. Eye Res..

[20] Parmet S., Lynm C., Glass R.M. (2006). Age-related macular degeneration. JAMA.

[21] Plainis S., Tzatzala P., Orphanos Y., Tsilimbaris M.K. (2007). A modified ETDRS visual acuity chart for European-wide use. Optom. Vis. Sci..

[22] Springer C., Bültmann S., Völcker H.E., Rohrschneider K. (2005). Fundus Perimetry with the Micro Perimeter 1 in Normal Individuals: Comparison with Conventional Threshold Perimetry. Ophthalmology.

[23] Ferris F.L., Kassoff A., Bresnick G.H., Bailey I. (1982). New visual acuity charts for clinical research. Am. J. Ophthalmol..

[24] Falkenstein I.A., Cochran D.E., Azen S.P., Dustin L., Tammewar A.M., Kozak I., Freeman W.R. (2008). Comparison of Visual Acuity in Macular Degeneration Patients Measured with Snellen and Early Treatment Diabetic Retinopathy Study Charts. Ophthalmology.

[25] Schuchard R.A. (1993). Validity and Interpretation of Amsler Grid Reports. Arch. Ophthalmol..

[26] Amsler M. (1953). Earliest symptoms of diseases of the macula. Br. J. Ophthalmol..

[27] Yoshimura N. (2013). Oct-Atlas.

[28] Patel P.J., Chen F.K., Ikeji F., Xing W., Bunce C., Da Cruz L., Tufail A. (2008). Repeatability of Stratus Optical Coherence Tomography Measures in Neovascular Age-Related Macular Degeneration. Investig. Opthalmol. Vis. Sci..

[29] Thomas D., Duguid G. (2004). Optical coherence tomography—A review of the principles and contemporary uses in retinal investigation. Eye.

[30] Macular Degeneration|Lake Travis Eye & Laser Center. https://laketraviseyecenter.com/macular-degeneration/.

[31] Trevino R. (2008). Recent progress in macular function self-assessment. Ophthalmic Physiol. Opt..

[32] Collazo E. (2011). Portable Electronic Amsler Test. U.S. Patent.

[33] Hirji R. (2008). Near Eye Opthalmic Device. U.S. Patent.

[34] Roser M.C. (2010). Visual and Memory Stimulating Retina Self-Monitoring System. U.S. Patent.

[35] Dowling J.E. (1987). The Retina: An. Approachable Part of the Brain.

[36] Sadun A.A., Wall M. (1989). System and Method of Detecting Visual Field Defects. U.S. Patent.

[37] Fink W., Sadun A.A. (2004). Three-dimensional computer-automated threshold Amsler grid test. J. Biomed. Opt..

[38] Loewenstein A., Malach R., Goldstein M., Leibovitch I., Barak A., Baruch E., Alster Y., Rafaeli O., Avni I., Yassur Y. (2003). Replacing the Amsler grid. Ophthalmology.

[39] Palanker D. (2014). Metamorphopsia Testing and Related Methods.

[40] Kohn W., Klingshirn J.A. (2014). Characterization and Correction of Macular Distortion. U.S. Patent.

[41] Mohaghegh N., Zadeh E.G., Magierowski S. (2016). Wearable diagnostic system for age-related macular degeneration. Proceedings of the 38th Annual International Conference of the IEEE Engineering in Medicine and Biology Society (EMBC).

[42] Mohaghegh N., Ghafar-Zadeh E., Munidasa S., Magierowski S. (2017). Toward Age-related Macular Degeneration (AMD) Big Data: Hardware and software design and implementation. Proceedings of the IEEE 30th Canadian Conference on Electrical and Computer Engineering (CCECE).

[43] Mohaghegh N., Munidasa S., Ziho X., Owen Q., Magierowski S., Ghafar-Zadeh E. (2018). Age-Related Macular Degeneration Diagnostic Tools: Hardware and Software Development. Proceedings of the IEEE 61st International Midwest Symposium on Circuits and Systems (MWSCAS).

[44] Lakshminarayanan V., Enoch J.M. (1995). Vernier acuity and aging. Int. Ophthalmol..

[45] Kaas J.H., Krubitzer L.A., Chino Y.M., Langston A.L., Polley E.H., Blair N. (1990). Reorganization of retinotopic cortical maps in adult mammals after lesions of the retina. Science.

[46] Loewenstein A. (2007). The significance of early detection of age-related macular degeneration: Richard & Hinda Rosenthal Foundation lecture, The Macula Society 29th annual meeting. Retina.

[47] Lai Y., Grattan J., Shi Y., Young G., Muldrew A., Chakravarthy U. (2011). Functional and morphologic benefits in early detection of neovascular age-related macular degeneration using the preferential hyperacuity perimeter. Retina.

[48] Robison C.D., Jivrajka R.V., Bababeygy S.R., Fink W., Sadun A.A., Sebag J. (2011). Distinguishing wet from dry age-related macular degeneration using three-dimensional computer-automated threshold Amsler grid testing. Br. J. Ophthalmol..

[49] Loewenstein A., Pollack A., Schachat A. (2002). Results of a Multicentered, Masked Clinical Trial to Evaluate the Macular Computerized Psychophysical Test (MCPT) for Detection of Age-related Macular Degeneration (AMD). Investig. Ophtalmol. Vis. Sci..

[50] Arimura E., Matsumoto C., Nomoto H., Hashimoto S., Takada S., Okuyama S., Shimomura Y. (2013). M-charts as a tool for quantifying metamorphopsia in age-related macular degeneration treated with the bevacizumab injections. BMC Ophthalmol..

[51] Inami Ltd. (2015). Quantitatable Metamorphopsia Chart. http://www.inami.co.jp/english/surgical_instruments/innovations/kdm1.

[52] Arimura E., Matsumoto C., Nomoto H., Hashimoto S., Takada S., Okuyama S., Shimomura Y. (2011). Correlations between M-CHARTS and PHP findings and subjective perception of metamorphopsia in patients with macular diseases. Investig. Ophtalmol. Vis. Sci..

[53] Wang Y.-Z., Wilson E., Locke K.G., Edwards A.O. (2002). Shape discrimination in age-related macular degeneration. Investig. Ophtalmol. Vis. Sci..

[54] Enoch J.M., Knowles R.A. (1989). Method for Evaluating Metamorphopsia. U.S. Patent.

[55] Wiecek E., Lashkari K., Dakin S., Bex P.J. (2014). Novel Quantitative Assessment of Metamorphopsia in Maculopathy. Investig. Ophtalmol. Vis. Sci..

[56] Stewart J.L. (2004). System and method for full field oscillating stimulus perimeter. U.S. Patent.

[57] Nazemi P.P., Fink W., Sadun A.A., Francis B., Minckler D. (2007). Early detection of glaucoma by means of a novel 3D computer-automated visual field test. Br. J. Ophthalmol..

[58] Faes L., Bodmer N.S., Bachmann L.M., Thiel M.A., Schmid M.K. (2014). Diagnostic accuracy of the Amsler grid and the preferential hyperacuity perimetry in the screening of patients with age-related macular degeneration: Systematic review and meta-analysis. Eye.

[59] Cocce K.J., Stinnett S.S., Luhmann U.F., Vajzovic L., Horne A., Schuman S.G., Toth C.A., Cousins S.W., Lad E.M. (2018). Visual Function Metrics in Early and Intermediate Dry Age-related Macular Degeneration for Use as Clinical Trial Endpoints. Am. J. Ophthalmol..

[60] Bennett A.G., Rabbetts R.B. (1989). Proposals for new reduced and schematic eyes. Ophthalmic Physiol. Opt..

[61] Kitzmann A.S., Pulido J.S., Diehl N.N., Hodge D.O., Burke J.P. (2008). The incidence of central serous chorioretinopathy in Olmsted County, Minnesota, 1980–2002. Ophthalmology.

[62] Crossland M., Rubin G. (2007). The Amsler chart: Absence of evidence is not evidence of absence. Br. J. Ophthalmol..

[63] Notal Vision Inc. (2015). ForeseeHome. http://www.foreseehome.com.

[64] Chew E.Y., Traci E.C., Susan B.B., Michael J.E., Ronald P.D., Jeffrey S., HeierfJudy E.K., Richard A.G. (2014). Randomized trial of the ForeseeHome monitoring device for early detection of neovascular age-related macular degeneration. The HOme Monitoring of the Eye (HOME) study design—HOME Study report number 1. Contemp. Clin. Trials.

[65] Han D.P. (2014). The foreseehome device and the home study: A milestone in the self-detection of neovascular age-related macular degeneration. JAMA Ophthalmol..

[66] How the ForeseeHome AMD Monitoring Program Works. https://www.foreseehome.com/hcp/how-it-works-technology/.

[67] Compass-Fundus Automated Perimetry. https://www.centervue.com/products/compass/.

[68] Miller K.P., Fortun J.A. (2018). Home Monitoring for Age-related Macular Degeneration. Curr. Ophthalmol. Rep..

[69] Schmid M.K., Faes L., Bachmann L.M., Thiel M.A. (2018). Accuracy of a Self-monitoring Test for Identification and Monitoring of Age-related Macular Degeneration: A Diagnostic Case-control Study. Open Ophthalmol. J..

